# CDC42-mediated Wnt signaling facilitates odontogenic differentiation of DPCs during tooth root elongation

**DOI:** 10.1186/s13287-023-03486-2

**Published:** 2023-09-19

**Authors:** Tao Zhou, Guoqing Chen, Yuchan Xu, Shuning Zhang, Huilin Tang, Tao Qiu, Weihua Guo

**Affiliations:** 1 State Key Laboratory of Oral Disease and National Clinical Research Center for Oral Diseases, West China Hospital of Stomatology, Sichuan University, Chengdu, China; 2 National Engineering Laboratory for Oral Regenerative Medicine, West China Hospital of Stomatology, Sichuan University, Chengdu, China; 3 Department of Pediatric Dentistry, West China Hospital of Stomatology, Sichuan University, Chengdu, China; 4 Frontier Innovation Center for Dental Medicine Plus, West China Hospital of Stomatology, Sichuan University, Chengdu, China; 5 School of Medicine, University of Electronic Science and Technology of China, Chengdu, China

**Keywords:** Odontogenesis, Tooth root, CDC42 GTP binding protein, Mesenchymal stem cells, Wnt signaling pathway

## Abstract

**Background:**

CDC42 is a member of Rho GTPase family, acting as a molecular switch to regulate cytoskeleton organization and junction maturation of epithelium in organ development. Tooth root pattern is a highly complicated and dynamic process that dependens on interaction of epithelium and mesenchyme. However, there is a lack of understanding of the role of CDC42 during tooth root elongation.

**Methods:**

The dynamic expression of CDC42 was traced during tooth development through immunofluorescence staining. Then we constructed a model of lentivirus or inhibitor mediated *Cdc42* knockdown in Herwig’s epithelial root sheath (HERS) cells and dental papilla cells (DPCs), respectively. Long-term influence of CDC42 abnormality was assessed via renal capsule transplantation and in situ injection of alveolar socket.

**Results:**

CDC42 displayed a dynamic spatiotemporal pattern, with abundant expression in HERS cells and apical DPCs in developing root. Lentivirus-mediated *Cdc42* knockdown in HERS cells didn’t disrupt cell junctions as well as epithelium-mesenchyme transition. However, inhibition of CDC42 in DPCs undermined cell proliferation, migration and odontogenic differentiation. Wnt/β-catenin signaling as the downstream target of CDC42 modulated DPCs’ odontogenic differentiation. The transplantation and in situ injection experiments verified that loss of CDC42 impeded root extension via inhibiting the proliferation and differentiation of DPCs.

**Conclusions:**

We innovatively revealed that CDC42 was responsible for guiding root elongation in a mesenchyme-specific manner. Furthermore, CDC42-mediated canonical Wnt signaling regulated odontogenic differentiation of DPCs during root formation.

**Supplementary Information:**

The online version contains supplementary material available at 10.1186/s13287-023-03486-2.

## Background

Tooth root is a multi-layer structure composed of dental pulp, dentin, cementum and periodontal ligament. The root mainly transmits occlusal force and serves as a channel for nutrition and sensation [1]. In addition, it helps to maintain adjacent bone homeostasis. Tooth root development occurs after the completion of crown formation. The inner enamel epithelium (IEE) and outer enamel epithelium (OEE) in cervical loop form a two-layered epithelial structure termed Herwig’s epithelial root sheath (HERS), which initiates the root development [2]. Then HERS continues to migrate downward to shape the root, and adjacent dental papilla cells (DPCs) and dental follicle cells (DFCs) differentiate into matrix-producing odontoblasts and cementoblasts. Multiple signaling pathways like wingless (Wnt), hedgehog (Hh) and bone morphogenetic protein (Bmp) interact to orchestrate normal tooth root morphogenesis [3]. However, the developing root is susceptible to endogenous and exogenous factors, leading to root dysplasia characterized in short root, absent furcation or curved roots [3–5]. To date, we have poor knowledge about the potential mechanism.

Cell division cycle 42 (CDC42) is a member of Rho family small GTPase. In 1990, John Pringle identified its role in yeast cell polarization for the first time [6]. CDC42 is then firmly established as a ubiquitous and evolutionarily conserved regulator involved in development, cancer, inflammation diseases and aging [7]. Recent cases propose that the clinical phenotypes of CDC42 mutation share some common features with Takenouchi-Kosaki syndrome, presenting with facial dysmorphism, neurodevelopmental delay, cardiovascular defects, musculoskeletal anomalies, and, in particular, severe immunodeficiency [8]. In animal models, loss of CDC42 can impair the development of kidney, heart, skin, bone and cartilage etc. [9–13]. During tooth development, CDC42 is abundantly expressed in epithelium in embryogenesis, and *Cdc42*-deletion in epithelium causes enamel organ cystogenesis and prevents successional dentin formation [14]. Another research indicates that mice with epithelium-specific *Cdc42* deletion have normal tooth phenotypes except for severe tooth attrition and reduced enamel volume [15]. This inconsistence might be attributed to the different time points of observation. Therefore, the role of CDC42 in tooth is complex and it’s unclear whether CDC42 modulates tooth root development.

Here, we mapped the expression of CDC42 and discovered that CDC42 was involved in tooth development from embryo stage to root development. Moreover, CDC42 was essential to regulate root elongation. We identified that cell junctions of HERS cells were not significantly impaired when CDC42 was knockdown in vitro experiments. But reduced CDC42 expression in DPCs evidently impaired cell proliferation, migration and odontogenic differentiation. Additionally, CDC42 regulated actin arrangement to control cell migration and collaborated with Wnt/β-catenin signaling to facilitate the differentiation of DPCs.

## Methods

### Cell isolation and culture

Animals (C57BL/6J mice and Sprague Dawley rats) were purchased from Dashuo Experimental Animal Co., Ltd. (Chengdu, China). The dental papilla was separated from first molar tooth germ of neonatal C57 mouse and HERS was obtained from the molar of P7 SD rat with micro forceps. Then they were digested in containing type I collagenase (3 mg/ml, Sigma, USA) for 30 min at 37 °C. Single-cell suspensions and tissue fragments were seeded and cultured at 37 °C with 5% CO2 in epithelial cell medium (EpiCM, STEMCELL Technologies, USA). The second passage cells were used in this study.

### Histology, immunohistochemical and immunofluorescence staining

All samples were fixed with 4% paraformaldehyde overnight at 4 °C, then decalcified in 10% EDTA (pH 7.0) at 37 °C for 2 weeks and embedded in paraffin. Tissue sections were prepared at 5 μm. They were stained with hematoxylin and eosin according to the instructions (Solarbio, China). For immunohistochemical/immunofluorescence staining, samples were blocked and incubated with primary antibodies overnight at 4 °C and cross-reacted with the secondary antibody about 1 h at room temperature. Immunocomplexes were visualized using a DAB reagent kit (Gene Tech, China). Primary antibodies included CDC42 (1:200, Abcam), Occludin (1:200, Abcam), E-cadherin (1:200, Zenbio), DMP1 (1:200, Novus Biologicals), COL-1 (1:200, abcam), RUNX2 (1:200, Huabio), β-catenin (1:200, Huabio), Ki67 (1:500, Huabio), Phalloidin (1:400, Abcam). The secondary antibodies included Alexa FluoR 555 goat anti-rabbit (1:400, Invitrogen), Alexa FluoR 555 goat anti-mouse (1:400, Invitrogen). Confocal images were acquired using a Leica STELLARIX5 microscope (Germany) and the following objectives: HC PL APO CS2 10×/0.40 DRY objective, HC PL APO 40×/0.95 DRY objective and HC PL APO 100×/1.44 OIL objective (Resolution: 1024 × 1024 square pixls). The laser lines were 555 nm and 405 nm, with the maximum intensity of 20%, and HyD detector gain 100%. Immunohistochemistry pictures were taken by Olympus IX73 (Japan) with 4×, 10× and 20× objectives (BF; Resolution: 1600 × 1200 square pixls). Red–green–blue images were assembled using Fiji software.

### Cdc42 lentivirus transfection

Lentivirus for Cdc42 knockdown was synthesized by OBiO Co., Ltd. (Shanghai, China). Four sequences of lentivirus were provided, including negative control without silencing effect and three sequences of Cdc42-target. The knockdown efficiency was tested by qRT-PCR and western blot.

### qRT-PCR

DPCs were cultured in 12-well plates and stimulated with 5 μM, 10 μM of ML141 (Absin, China) for two days. DPCs cultured in α-MEM serving as blank control during osteogenic induction. Total RNAs were extracted using the FastPure cell/tissue total RNA isolation kit (Vazyme, China). After extraction, 500 ng–1 μg of total RNAs were reverse transcribed to cDNA using HiScript® III RT Super Mix (Vazyme, China). qRT-PCR was conducted using Taq pro Universal SYBR qPCR Master Mix (Vazyme, China) in a LightCycler480 system (Roche, Sweden). The relative expression levels were calculated with the comparative threshold cycle (ΔΔCT) method.

### Western blotting and pull-down assay

DPCs were collected and lysed using RIPA buffer (KeyGEN, China) for 30 min at 4 °C. The nuclear protein was collected using cytoplasmic and nuclear extraction kit (Invent, USA). The protein lysates were quantified by BCA Protein Assay Kit (KeyGEN, China). Equal 15–20 μg protein extracts were loaded per lane. After primary antibody and PHRP-conjugated secondary antibody incubation, the protein bands were visualized using BLT GelView 6000Plus system (BLT, China). For pull-down assay, CDC42 activity was tested according to the CDC42 activation kit protocol (NewEastBiosciences, USA). Antibodies included CDC42 (1:1000, abcam), DMP1 (1:1000, Biovision), DSPP (1:1000, Zenbio), ALP (1:1000, Huabio), p-GSK3β (1:1000, Huabio), β-catenin (1:1000, Huabio), Col-1 (1:1000, abcam), GAPDH (1:5000, abcam), Occludin (1:1000, Abcam), E-cadherin (1:1000, Zenbio).

### CCK8 and transwell migration assay

The viability of DPCs was measured by Cell Counting Kit-8 (KeyGEN, China) according to the manufacturer’s protocol. DPCs were seeded in 96-well plates at a density of 1 × 10^4^ cells/well with gradients of 5 uM, 10 uM, 15 uM, 20 uM of ML141 at different time points (12 h, 1d, 2d, 3d, 6d). CCK8 reagents were added and incubated with cells for 2 h at 37 °C, then the absorbance at 450 nm was detected using a spectrophotometer (Thermo Fisher Scientific Inc, USA). 4 × 10^4^ cells were seeded on the upper chamber with 8.0um pore membrane (LABSELECT, China). After 24 h, 200ul 1% crystal violet solution (Biosharp, China) was added in every chamber to stain for 1 h. We viewed five different fields underneath a Leica DMi8 microscope (Germany) with 10× objective (BF; Resolution: 1824 × 1216 square pixels). Then we counted the number of cells to get an average sum of cells using Fiji software. The crystal violet was dissolved by 33% glacial acetic acid and quantified by absorbance at 570 nm.

### EdU assay and live and dead staining

2 × 10^4^ cells per well were seeded in 96-well plates for 24 h, then the cells were incubated with different concentrations of ML141 (5 μM, 10 μM, 15 μM, 20 μM) for another 24 h. For Edu assay, cells were incubated with 50 μM EdU solution for 2 h then were stained according to the protocol of Cell-Light™ EdU Apollo In Vitro Kit (Ribobio, China). Three different fields were imaged and counted to get an average sum of cells. For live and dead staining, cells were washed with PBS gently, then followed by addition of 2 uM Calcein-AM**/**8 uM Propidium-iodide (KeyGEN, China) at room temperature for 30 min. The stained cells were observed via Olympus IX73 microscope (Japan) using 20×/PH1 objective (U-FUNA: DAPI; F-FBNA: FITC; Resolution: 1600 × 1200 square pixels). Images were assembled using Fiji software.

### Alizarin red staining (ARS) and alkaline phosphatase (ALP) staining

To detect osteogenic differentiation ability, DPCs were cultured in osteogenic medium (OM, α-MEM supplemented with 10% FBS, 5 mM l-glycerophosphate, 100 nM dexamethasone, and 50 μg/ml ascorbic acid) in 12-well plates. After 10 days induction, the cells were fixed using 4% paraformaldehyde for 30 min and calcium deposition were stained by 2% ARS. ALP was detected at day4 after induction by using BCIP/NBT Alkaline Phosphatase Chromogenic Kit (Beyotime, China). ALP activity was measured by an ALP activity kit (Jiancheng, China) and the absorbance of each well was determined by measurements at 520 nm.

### In vivo kidney capsule transplantation and in situ injection

The mandibular first molar germs dissected from P3 C57BL/6J mice were used for transplantation. A total of 12 male C57BL/6J mice of 8 weeks were randomly divided into three groups (Control, PBS and ML141), with 4 individuals in each. The blue beads (GE healthcare biosciences, USA) were incubated with PBS and ML141 one day ahead. Then the tooth germs and beads were transplanted into the left renal capsule of the mouse. After 4 weeks, the grafts were obtained and fixed to perform micro-CT and histological analysis.

A total of 16 Postnal7 (P7) SD male rats were randomly divided into three groups, Blank (*n* = 4), PBS (*n* = 4) and ML141 (*n* = 8). They were anesthetized and injected around 2 mm behind the second palatine rugae toward the bilateral maxillary first molar germ lingual direction with 2 mm depth. 10 uM ML141 were conducted using a micro-syringe (Hamilton, Sweden) with 8ul every other day for long-term inhibiting experiments until P16.

Animals were kept under standardized conditions (temperature: 20–26 °C; relative humidity: 10–70%; ammonia concentration: ≤ 14 mg/m^3^; cleanliness class 7; ≤ 60 dB; a 12 h light cycle), with free access to formula feeds and water. Dislocation of cervical vertebra was utilized in C57BL/6J mice euthanasia, since it was simple to do without endangering visceral organs and could instantly relieve discomfort. Pentobarbital (150 mg/kg) was administered intraperitoneally to euthanize SD rats, and this procedure was quick, efficient and affordable. All animal procedures complied with the guidelines of the ARRIVE 2.0 checklist.

### Micro-CT scanning and analysis

All samples were scanned using a Quantum GX micro-CT system (PerkinElmer, USA). The samples were scanned under the following conditions: acquisition: 36; recon: 10; voxel size: 10.0 μm; scan mode: high resolution for imaging of the transplanted drafts or maxillary bone. Morphologic observation and measurements were assessed using Quantum GX software. Three-dimensional (3D) reconstruction was done with Mimics. We carefully defined the root length methods, which started from enamel-cementum junction and ended at apical foramen in sagittal section. All measurements were performed by one analyst.

### Statistical analysis

All data were presented as mean ± standard deviation (SD) and analyzed using GraphPad Prism. Statistical significance was assessed by two-tailed Student’s t-test and one-way ANOVA for multiple comparison. Fiji software was used for quantification analysis of immunohistochemical/immunofluorescence staining. At least three technical replicates were measured and quantified. For in vivo study, we chose samples from at least three individual mouse and selected representative images. *P* < 0.05 was considered to be statistically significant.

## Results

### The spatiotemporal expression of CDC42 during tooth development

**Fig. 1:**
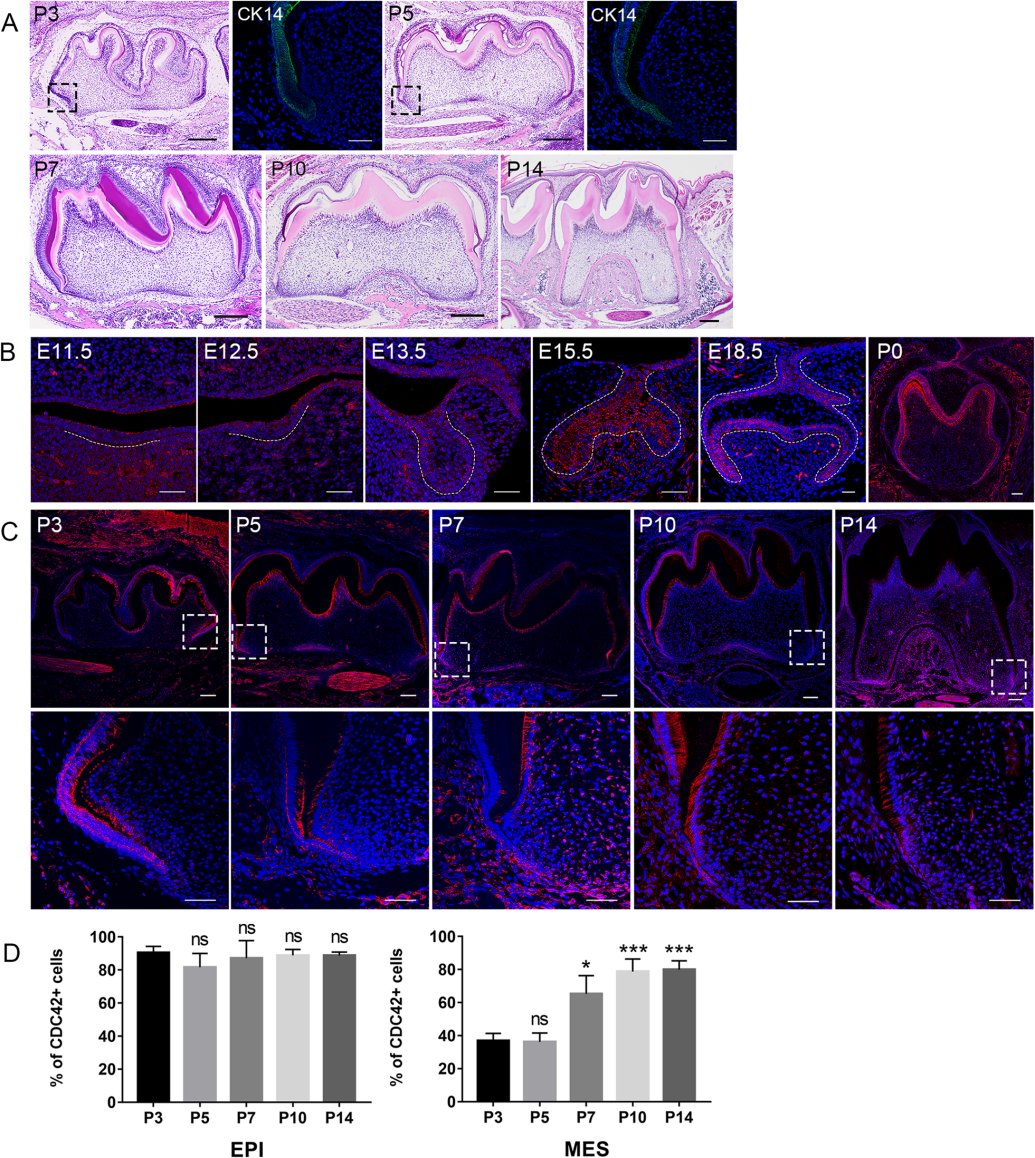
CDC42 expressed in the epithelium and adjacent mesenchyme during tooth development. **A** Different stages of tooth root development. The change of epithelium in P3 and P5 was indicated by CK14. The black square showed the magnification of the cervical loop. **B** The expression of CDC42 in embryonic stage. Dotted lines indicated the border between dental epithelium and mesenchyme. **C** Immunofluorescence indicated that CDC42 expressed in HERS, odontoblasts and apical DPCs in developing root. The white square showed the magnification of the apical area of root. **D** Quantification analysis of CDC42 expression from P3 to P14. Scale bars: 50 μm (**A–C**), 100 μm (**C**), 200 μm (**A**). Data are presented as mean ± SD. *p < 0.05, ***p < 0.001, *ns* no significance

Tooth development commenced at around embryonic (E)11.5, then it experiences the bud (E13.5), cap (E15.5), early bell (E16.5) and late bell stage (E18.5) to shape the enamel organ. The tooth crown was completed at P3 and then tooth root development started (Fig. 1A). At stage of root initiation, multilayer cervical loop cells transformed into bilayer HERS cells, which was illustrated by CK14 (Fig. 1A). As HERS extended downward, the tooth root elongated rapidly from P7 to P14 (Fig. 1A). Then we mapped the expression of CDC42 from dental lamina stage to root elongation stage. From E11.5 to E13.5, the expression was modest and non-specific. As the polarity arose in enamel epithelium, it robustly expressed in dental epithelium and adjacent mesenchyme, especially in IEE and OEE (Fig. 1B). The expression of CDC42 attenuated in stellate reticulum (SR) and stratum intermedium (SI) from E15.5 to E18.5 (Fig. 1B), while it sustained a high level in enamel epithelium from embryonic stage to root development. Concurrently, it was detectable in HERS, odontoblasts and apical dental papilla from P5 (Fig. 1C). As the root extended downward, the expression of CDC42 in apical dental papilla significantly increased (Fig. 1C, D). The robust expression in ameloblasts and odontoblasts may be attributed to the crucial role of CDC42 in highly polarized cells [16–18]. However, the function of CDC42 in HERS cells and DPCs are poorly studied.

### HERS-specific *Cdc42* knockdown had little impact on cell junctions and epithelium-mesenchyme transition

**Fig. 2:**
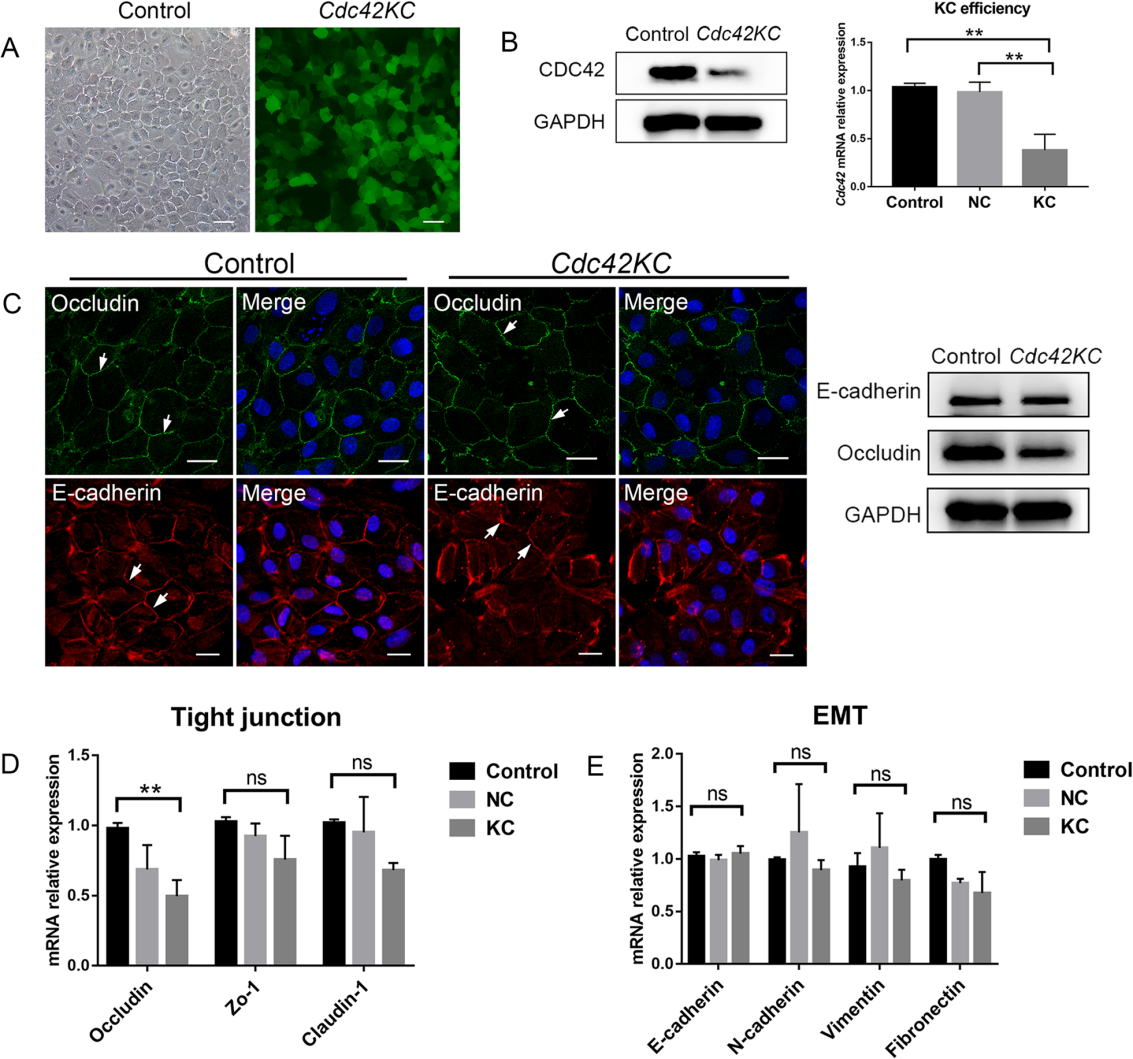
*Cdc42* knockdown in HERS cells didn’t impair cell junctions and epithelium-mesenchyme transition. **A** Lentivirus-transfected HERS cells were labeled with GFP. **B** Western blot and qRT-PCR showed the effective inhibition of *Cdc42* expression (Full-length blots are presented in Additional file 2). **C** Western blot and confocal images of tight junction (green) and adhesive junction (red). The arrows indicated connections on cell membrane (Full-length blots are presented in Additional file 2). **D** Quantification of tight junction relative genes including *Occludin*, *Zo-1*, *Claudin-1* in two groups. **E** Quantification of epithelium-mesenchyme transition relative genes including *E-cadherin*, *N-cadherin*, *Vimentin*, *Fibronectin* in two groups. Scale bars: 50 μm (**A**), 20 μm (**B**). Data are presented as mean ± SD. **p < 0.01, *ns* no significance

HERS is regarded as the pivotal member to initiate root development. As for the abundant expression of CDC42 on HERS, we constructed a lentiviral transfection model of HERS-specific knockdown of *Cdc42*. HERS cells showed cobblestone-like appearance and lentivirus-transfected cells were labeled with green fluorescent protein (GFP) (Fig. 2A). The expression of CDC42 evidently decreased when MOI = 20. qRT-PCR further quantified the knockdown efficiency, which was approximately 70% (Fig. 2B, Additional file 2). HERS cells displayed tight connections with adjacent cells. Immunofluorescence staining indicated that the distribution of Occludin was similar in control and knockdown group. However, the level of Occludin reduced significantly when *Cdc42* was inhibited (Fig. 2C, Additional file 2). The distribution of E-cadherin in cell membrane was still contiguous and the expression of E-cadherin wasn’t affected in knockdown group (Fig. 2C, Additional file 2). During tooth root elongation, rear HERS cells experience the fragmentation of tight and adhesive junction, thus transforming into mesenchymal cells. Therefore, we quantified the tight junction and epithelium-mesenchyme transition (EMT) relative genes. After *Cdc42* was inhibited, the expression of *Occludin* was downregulated both at protein and mRNA level (Fig. 2C, D). As for *Zo-1*, *Claudin-1*, *E-cadherin*, *N-cadherin*, *Vimentin and Fibronectin*, there was no significance between control and knockdown group (Fig. 2D, E). This outcome supported that cell junctions and EMT of HERS cells were not prominently impaired after *Cdc42* was knockdown.

### Inhibition of CDC42 impaired DPCs proliferation, migration and odontogenic differentiation through Wnt/β-catenin signaling

**Fig. 3:**
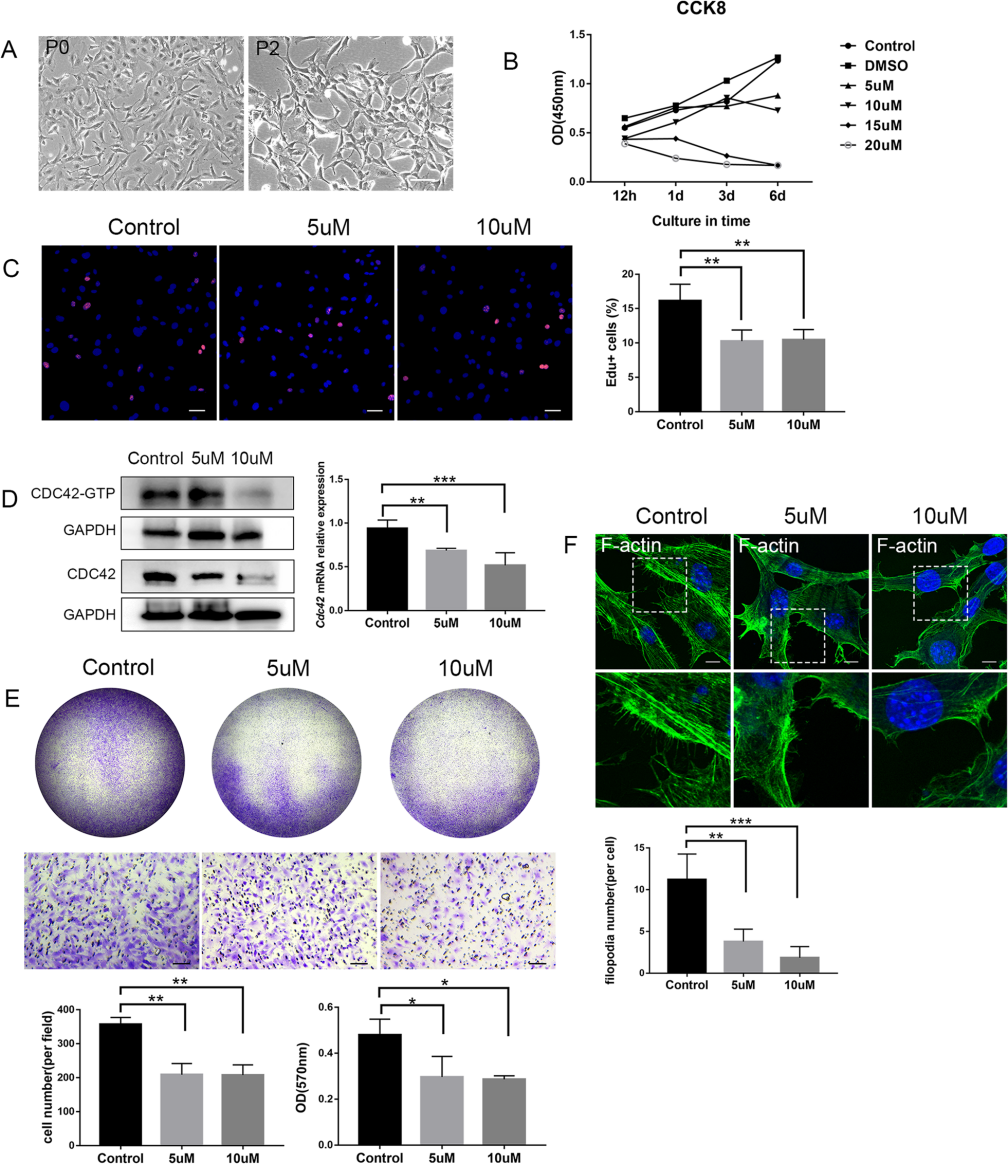
CDC42 was crucial for the proliferation and migration of DPCs. **A** Representative images of primary DPCs P0 and P2. **B** Influences of gradients of ML141 on the viability of DPCs at different time points. **C** EdU assay revealed the decreased ability of proliferation in 5 μM and 10 μM group. **D** ML141 effectively inhibited expression of CDC42 and CDC42 active form (Full-length blots are presented in Additional file 3). **E** Transwell assay and quantification indicated the hindered capability of migration in 5 μM and 10 μM group. **F** The number of filopodia decreased in 5 μM and 10 μM group. Scale bars: 100 μm (**A**, **E**), 50 μm (**C**), 10 μm (**F**). Data are presented as mean ± SD. *p < 0.05, **p < 0.01, ***p < 0.001

DPCs obtained from new born mice were typically spindle-shaped cells (Fig. 3A). ML141 is an effective inhibitor of CDC42 and is widely used [19]. CCK8 assay indicated that ML141 was adverse to cell viability, and the inhibiting effect got more apparent as the concentration increased (Fig. 3B). This outcome was consistent with the live and dead staining that DPCs obviously died at 15 μM and 20 μM (Additional file 1: Figure S1). Thus we chose 5 μM and 10 μM in following experiments. Western blot and qRT-PCR analysis revealed that ML141 can effectively inhibited both CDC42 total expression and CDC42-GTP form in DPCs (Fig. 3D, Additional file 3). To further evaluate the influence of ML141 on DPCs proliferation, we did EdU assay and found DPCs proliferation was inhibited at 5 μM and 10 μM (Fig. 3C). Transwell assay was conducted to explore the function of CDC42 in DPCs migration. There were less migrated DPCs in ML141-treated group compared with that in control group after 24 h (Fig. 3E). As filopodia providing driving force in cells migration, then we visualized the filopodia formation in migrated DPCs (Fig. 3F). The immunofluorescence and quantification analysis indicated that the number of filopodia decreased when CDC42 was suppressed, thus exhibiting inhibited ability of migration.

**Fig. 4:**
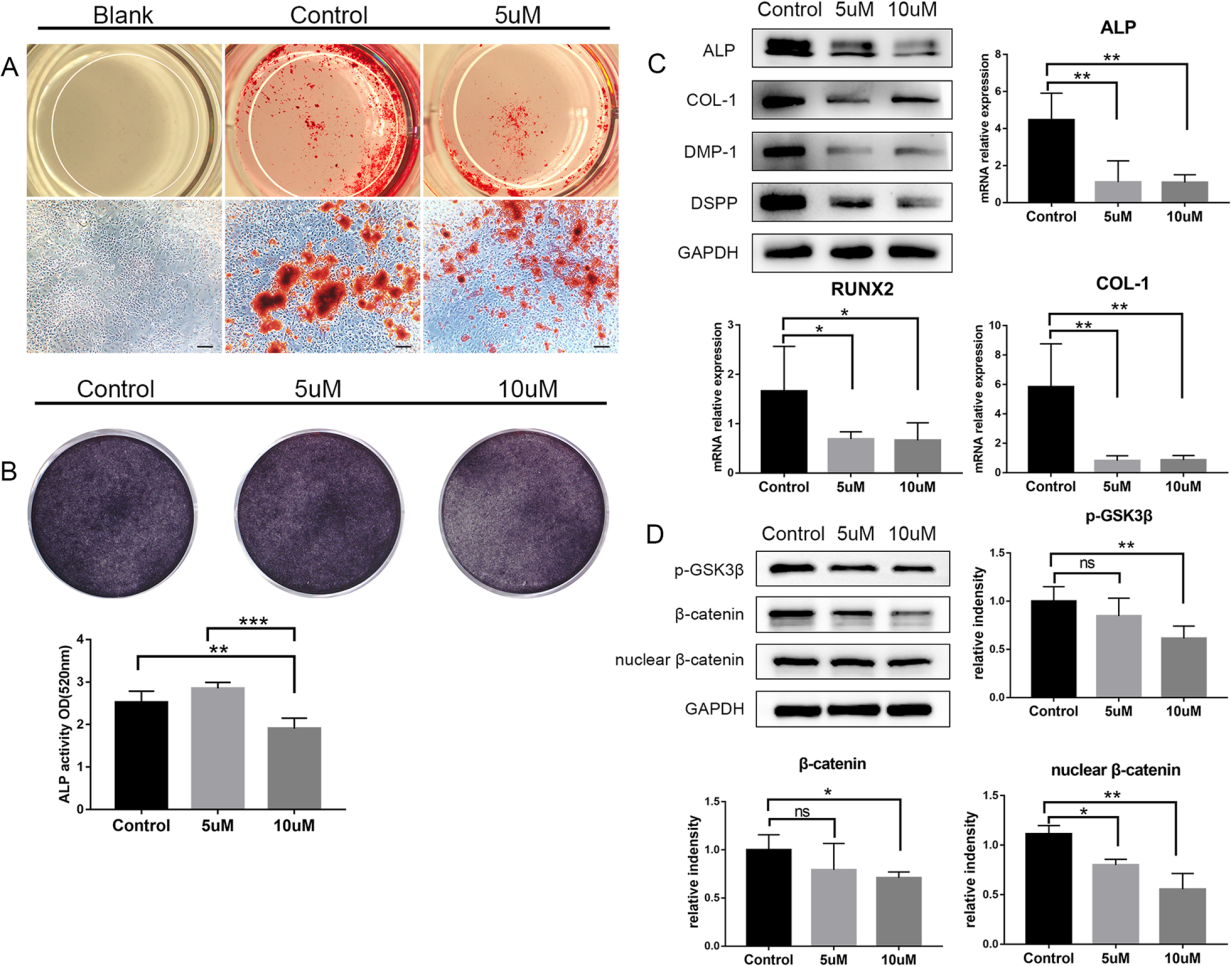
Inhibition of CDC42 disturbed odontogenic differentiation of DPCs. **A** Attenuated mineralization in 5 μM group. The calcium nodules were stained with red. **B** Alleviated ALP staining and ALP activity when CDC42 was inhibited. **C** Expression of odontogenic markers (ALP, COL-1, DMP-1, DSPP) were downregulated after treated with ML141 (Full-length blots are presented in Additional file 4). **D** Western blot and quantification suggested reduced phosphorylation of GSK-3β in cytoplasm and accumulation of β-catenin in nucleus (Full-length blots are presented in Additional file 4). Scale bars: 200 μm (**B**). Data are presented as mean ± SD. *p < 0.05, **p < 0.01, ***p < 0.001, *ns* no significance

To characterize the role of CDC42 in DPCs odontogenic differentiation, we cultured DPCs in osteo-induction medium. The positive control identified the osteogenic differentiation ability of DPCs in vitro according to ARS staining. The number of mineralized nodules reduced in 5 μM group compared with the control (Fig. 4A). In addition, the outcome of ALP staining and ALP activity measurements also supported the weakened odontogenic differentiation ability after treated with 10 μM ML141 (Fig. 4B). But the odontogenic differentiation ability of DPCs in the 5 μM group wasn’t influenced (Fig. 4B). Western blot revealed that osteo/odontogenic relative markers (ALP, COL-1, DMP-1, DSPP) were downregulated at protein level, with more obvious inhibition in 10 μM group (Fig. 4C, Additional file 4). The quantitative analysis indicated that the mRNA expression of ALP, RUNX2 and COL-1 reduced prominently both in 5 μM and 10 μM group, which was consistent with the western blot (Fig. 4B, C).

To further probe the molecular mechanism of CDC42-mediated signaling in DPCs, we analyzed the change of protein level of canonical Wnt pathway. The diminution of CDC42 in DPCs attenuated phosphorylation of GSK-3β, thus depressing accumulation of β-catenin in nucleus (Fig. 4D, Additional file 4). The depression efficiency was higher in 10 μM group according to the quantification analysis, which supported the obviously inhibited odontogenic differentiation at 10 μM.

### CDC42 is vital for tooth root elongation in renal capsule transplantation

**Fig. 5:**
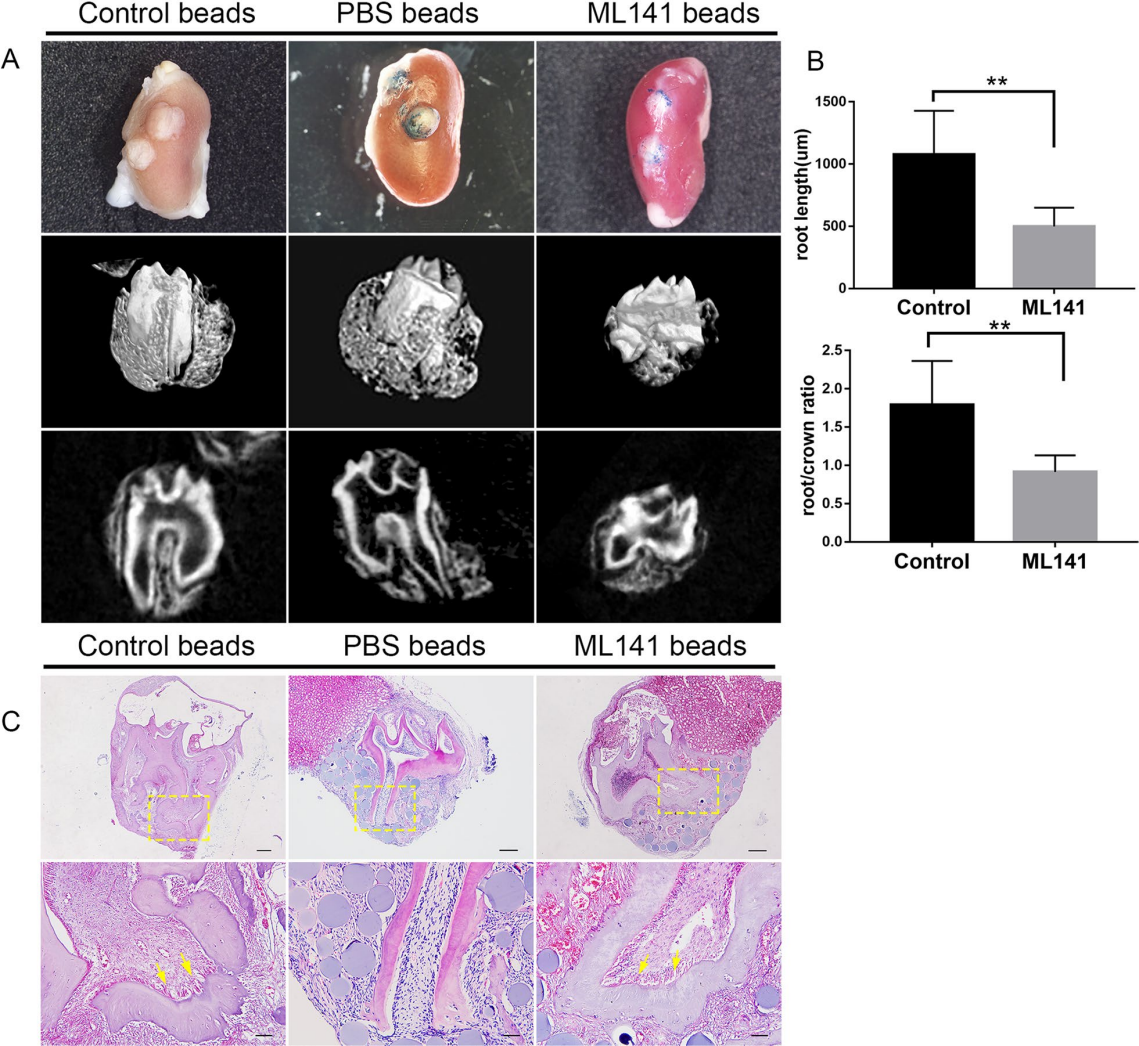
Tooth root elongation was impaired as a result of CDC42 abnormality. **A** After transplantation for 4 weeks, the control and PBS-soaked explants developed normal root structure and numbers (*n* = 4), while ML141-soaked explants behaved shortened roots (*n* = 4). **B** Quantification of the root length and ratio of root/crown. **C** HE analysis indicated shortened roots and aberrant odontoblasts after CDC42 was inhibited. The boxes showed the magnification of the apical region. The yellow arrowheads indicated less well-organized odontoblasts. Scale bars: 200 μm, 50 μm (**C**). Data are presented as mean ± SD. **p < 0.01

To explore whether CDC42 influences tooth root development in vivo, we performed heterotopic renal capsule transplantation which can provide blood supply for organ development. Expectedly, all tooth germs continued to develop in the kidney, exhibiting tooth volume increase, tooth root elongation, as well as the alveolar bone and consecutive periodontal ligament formation (Fig. 5A). Inhibition of CDC42 resulted in shortened roots while the root diameter and number were unaffected (Fig. 5A). Statistical analysis showed that root length in the ML141 group decreased compared to the control group, and the root/crown ratio were less than 1:1 (Fig. 5B). And the root length in ML141 group was around half of that in control group. Furthermore, we observed that periodontal ligament was irregular and multidirectional in all groups. Strikingly, in the apical region, ML141 group displayed less well-organized and scattered odontoblasts (Fig. 5C).

### Continuous inhibition in situ impeded root elongation via disrupting apical DPCs proliferation and differentiation

**Fig. 6:**
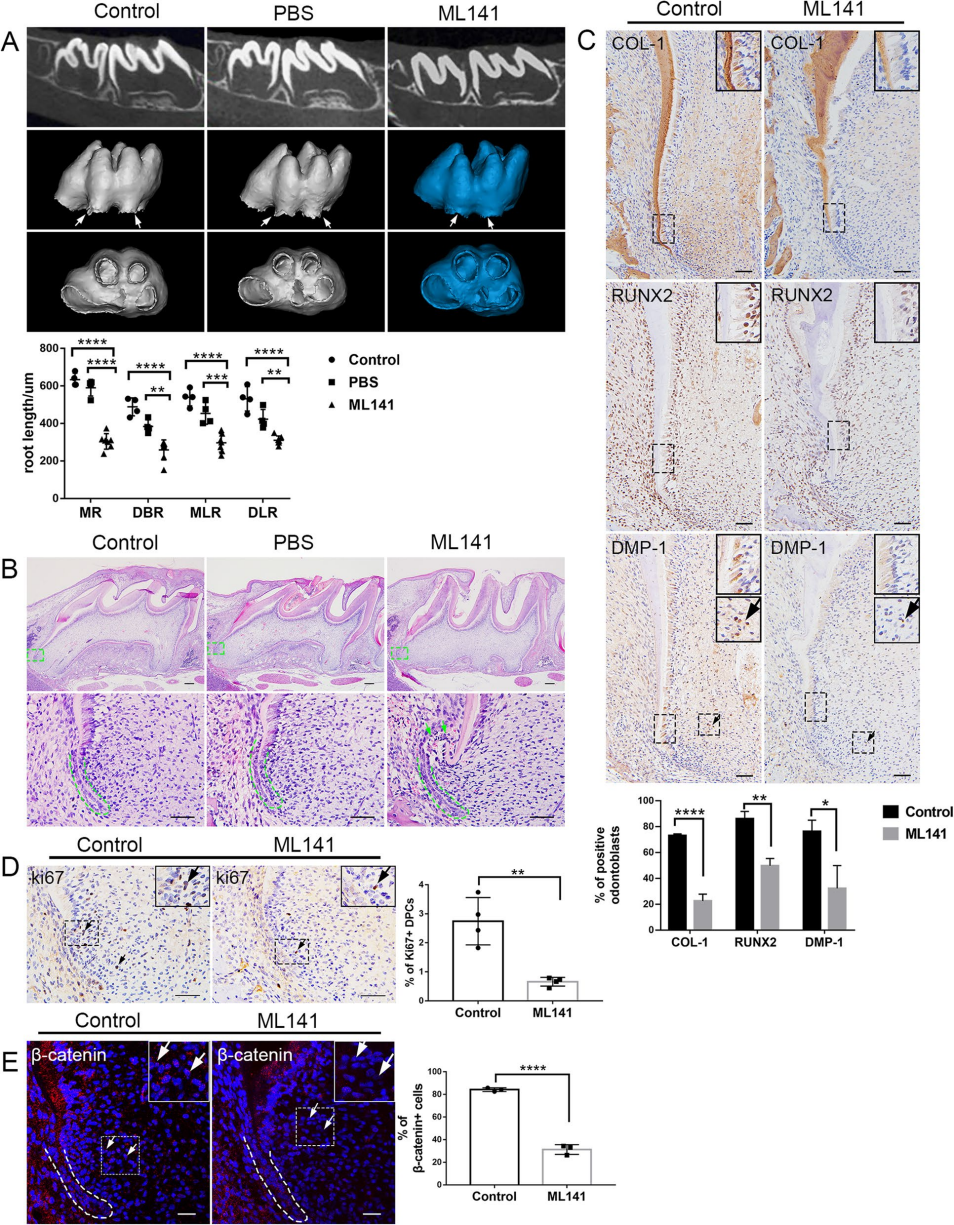
ML141 impeded root development through inhibiting the proliferation and differentiation of DPCs in apical region. **A** Micro CT analysis and quantification indicated that ML141 impeded root development of MR, DBR, MLR and DLR (Control, *n* = 4; PBS, *n* = 4; ML141, *n* = 8). **B** HE analysis displayed the shortened roots in ML141 group. The boxes showed the magnification of apical area. Dotted lines indicated the shape of HERS. Arrowheads showed the curved structure. **C** Immunohistochemical staining of COL-1, RUNX2 and DMP-1 and quantification analysis. The arrowheads indicated DMP-1 positive DPCs. **D** Immunohistochemical staining and quantification of Ki67 + DPCs. The arrowheads indicated ki67 + DPCs. **E** Immunofluorescence staining and quantification of β-catenin in apical region. The arrowheads showed β-catenin expression of DPCs. Scale bars: 200 μm (**B**), 50 μm (**B**–**E**). Data are presented as mean ± SD. **p < 0.01, ***p < 0.001, ****p < 0.0001

Tooth socket possesses specific environment which is beneficial to tooth root development [20]. To explore the functional significance of CDC42-mediated signaling during root elongation, we conducted in situ injection of alveolar socket around first molar every other day from P7 to give a continuous inhibition. Analogous to that in renal capsule transplantation, tooth root development was significantly blocked at PN16 after CDC42 activity was disturbed, behaving short root anomaly (Fig. 6A). We then measured the root length of mesial root (MR), distal buccal root (DBR), mesial lingual root (MLR) and distal lingual root (DLR) of the maxillary first molar. Although PBS group behaved shorter roots than control group, there was no significance in MR and MLR (Fig. 6A). Obviously, ML141 group displayed impeded root elongation (Fig. 6A), and the root formation was not even observed in one group. HE analysis revealed no impairment of the bilayered structure of HERS and closely arranged HERS cells (Fig. 6B). There was a curved structure between HERS and apical mineralization area in several samples (Fig. 6B). Moreover, the expression of COL-1, RUNX2 and DMP-1 decreased in ML141 group. Both the expression of COL-1 and DMP-1 reduced in odontoblasts and DPCs. RUNX2 expressed widely in alveolar bone, dental follicle, periodontal ligament and apical dental papilla during developing root. And its expression also declined in odontoblasts in ML141 group (Fig. 6C). A significant reduction of DPCs proliferation in root apical area was also observed based on Ki67 immunostaining and Ki67 + count, partially explained the shortened root length at PN16 (Fig. 6D). We further mapped β-catenin expression at PN16 in vivo. HERS and DPCs both expressed high levels of β-catenin, indicating that the canonical Wnt signaling pathway was involved in tooth root development (Fig. 6E). After CDC42 was inhibited, the β-catenin expression of DPCs close to HERS was also downregulated (Fig. 6E). Therefore, CDC42 inhibition mainly delayed tooth root development through decreasing DPCs proliferation and differentiation.

## Discussion

In most organ development, CDC42-mediated apical cell polarity and junction stability control epithelium homeostasis. And the function diversity arises from that CDC42 can exert its function through many effectors, such as atypical PKC (aPKC) and neural Wiskott–Aldrich syndrome protein (N-WASP) [21]. The aberration of CDC42 disrupts epithelium morphogenesis such as branching, tubulogenesis in early embryo development, which causes development hypoplasia [17, 22]. In epithelium stem niche, CDC42-YAP-mTOR axis is pivotal to epithelium cells proliferation and differentiation, which has been confirmed in incisor and intestine [23, 24]. A recent study reported that enamel epithelium-specific *Cdc42* deletion caused disrupted actin assemblies, aberrant desmosomes, and reduced cell junctions, thus resulting in cystic lesions in the developing enamel organ [14]. In this study, we found CDC42 was expressed in HERS in developing root, specific knockdown of *Cdc42* in HERS cells didn’t impair cell junctions and EMT, which was different from that in enamel epithelium. And the bilayered structure of HERS was unaffected in ML141-treated group according to the HE analysis in vivo experiments. We attribute this difference to the fact that (1) HERS cells lack typical cell polarity unlike enamel epithelium. (2) HERS is a transitional structure in tooth development which possesses both of the epithelium and mesenchyme features. However, conditional knockout mice are required to study the specific role of *Cdc42* in HERS during tooth root development in further studies.

Recently, an ascendant number of researches account that CDC42 can interact with other pathways to regulate MSCs behavior. CDC42/PAK/p38/Smad signaling can promote mesenchyme condensation [25]. CDC42 deficiency downregulates MAPK1/3 and SOX9 expression, thus damaging spermatogonial stem cells maintenance [26]. Notably, Wnt signaling and CDC42 crosslink to regulate MSCs biological process. Elevated Wnt5A expression in aging hair follicle stem cells upregulates the expression of CDC42 [27]. CDC42 also interacts with canonical Wnt signaling to contribute bone mesenchymal stem cells’ osteoblastic differentiation in titanium surface, which provides a novel strategy in regeneration engineering [28]. In addition, CDC42 is vital to maintain the level of β-catenin to contribute to adipose mesenchymal stem cells to derived cells to secret insulin [29]. The expression of CDC42 was upregulated after sciatic nerve injury and CDC42-Wnt/β-catenin signaling was identified to promote cell proliferation [30]. Likewise, our study identified the importance of CDC42-Wnt/β-catenin pathway in regulating MSCs differentiation in tooth root development. The odontogenic differentiation capability of DPCs was disturbed evidently as a result of inhibition of CDC42. CDC42 belongs to the Rho GTPase family, a recent study also reported that loss of Rho guanine nucleotide exchange factor (Rho-GEP) disrupted root formation with influencing DPCs differentiation through p38 MAPK pathway [31].

Meanwhile, the front-rear polarity establishment and lamellipodia extension are implicated in mesenchyme stem cells migration [32]. CDC42 promotes actin reorganization as well as filopodia formation simultaneously to contribute cell migration, thus providing apical force for root elongation. Practically, CDC42 tends to accumulate in cell edge before migration [33] and is involved in filopodia formation acting through formins [34]. Our study also supported that CDC42 contribute to the number of filopodia to facilitate cell migration.

Tooth root as a supportive structure connects the bone and tooth crown to transmit occlusal force, thus the root length is particularly important. In clinic, patients with short root are more susceptible to root resorption during orthodontic treatment [35]. Canonical and noncanonical Wnt signaling are involved in tooth root formation, based on Axin2 and Wnt10a expression in odontoblasts and pre-odontoblasts [36, 37]. Our study also supported the participation of Wnt signaling in root development because of the expression of β-catenin in HERS cells and DPCs. Excessive or inhibited β-catenin in odontoblasts will influence odontoblasts differentiation, thus disturbing root formation [38, 39]. And it also cooperates with other signals like RUNX2, TGFβr2 to control pre-odontoblasts differentiation [40, 41]. Except for the mesenchyme, Wnt/β-catenin signaling also functions in HERS, depleted β-catenin in HERS cells results in the disruption of HERS adhesion and junction proteins, which can disrupt root formation [42]. Practically, Wnt/β-catenin signaling was not only involved in the process of odontoblasts matrix-producing stage, our study also identified the CDC42-Wnt/β-catenin signaling was required in the process of early DPCs odontogenic differentiation and proliferation. According to in vivo experiments, we considered that reduced β-catenin expression in DPCs was adverse to root elongation during early root development. This conclusion agrees to the opinion that root elongation is sensitive to β-catenin level within a narrow window of time, from new born to P5 [43].

## Conclusions

Taken together, we suggest CDC42 acting as the crucial mediator in DPCs proliferation, migration and odontogenic differentiation. Inhibition of CDC42 alleviates Wnt/β-catenin-mediated odontogenic genes expression in DPCs, thus causing short root anomaly.

### Supplementary Information

**Additional file 1**: Supplementary Figures.

**Additional file 2**: Original blot images of Figure S2B and Figure S2C.

**Additional file 3**: Original blot images of Figure S3D.

**Additional file 4**: Original blot images of Figure S4C and Figure S4D.

## Data Availability

The data that support the findings of this study are available from the corresponding author upon reasonable request.

## Abbreviations

DPCs: Dental papilla cells
IEE: Inner enamel epithelium
OEE: Outer enamel epithelium
HERS: Herwig’s epithelial root sheath
DFCs: Dental follicle cells
Wnt: Wingless
Hh: Hedgehog
Bmp: Bone morphogenetic protein
CDC42: Cell division cycle 42
SR: Stellate reticulum
SI: Stratum intermedium
EMT: Epithelium-mesenchyme transition
MR: Mesial root
DBR: Distal buccal root
MLR: Mesial lingual root
DLR: Distal lingual root
GFP: Green fluorescent protein
ALP: Alkaline phosphatase
ARS: Alizarin red staining
