# Supplementary material for: CDC42-mediated Wnt signaling facilitates odontogenic differentiation of DPCs during tooth root elongation
Source: Stem Cell Res Ther. 2023 Sep 19;14:255. doi: 10.1186/s13287-023-03486-2 (PMC10510226; doi:10.1186/s13287-023-03486-2)
Supplement: Supplementary file 1 — Additional file 1: Supplementary Figures. [file 13287_2023_3486_MOESM1_ESM.docx]

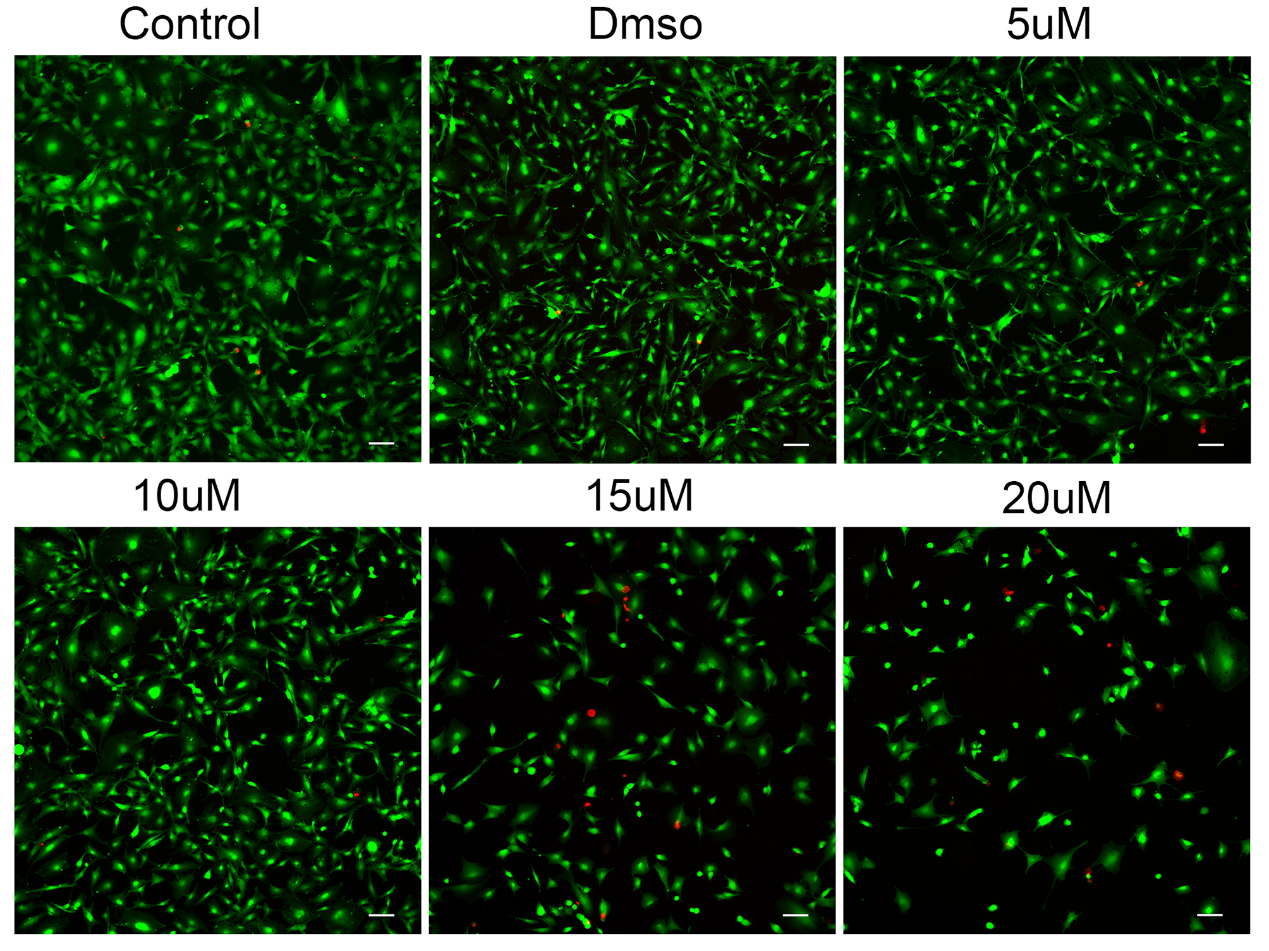


**Additional file1: FigureS1.** Live and dead staining of DPCs. The number of DPCs decreased and DPCs died evidently in 15uM and 20uM. 100μm
