# Supplementary material for: CDC42-mediated Wnt signaling facilitates odontogenic differentiation of DPCs during tooth root elongation
Source: Stem Cell Res Ther. 2023 Sep 19;14:255. doi: 10.1186/s13287-023-03486-2 (PMC10510226; doi:10.1186/s13287-023-03486-2)
Supplement: Supplementary file 3 — Additional file 3: Original blot images of Figure S3D. [file 13287_2023_3486_MOESM3_ESM.docx]

**Additional file3.** Original photos of the full bots in Figure3D

| CDC42-GTP: 21kda | 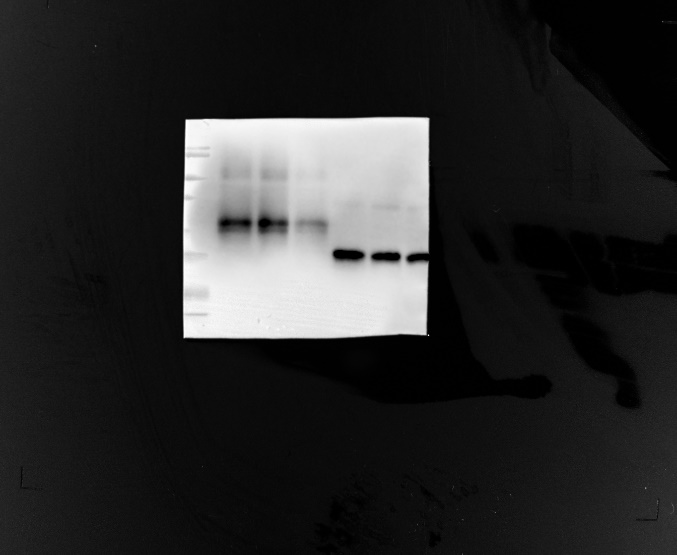 |
| --- | --- |
| GAPDH: 36kda | 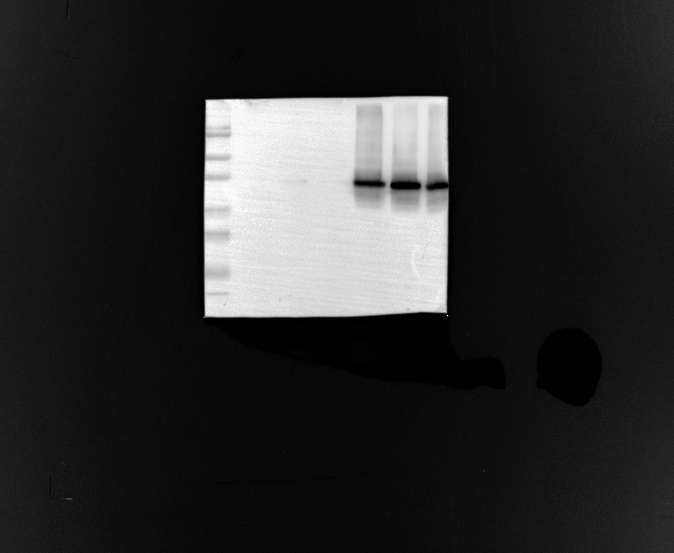 |
| CDC42: 21kda | 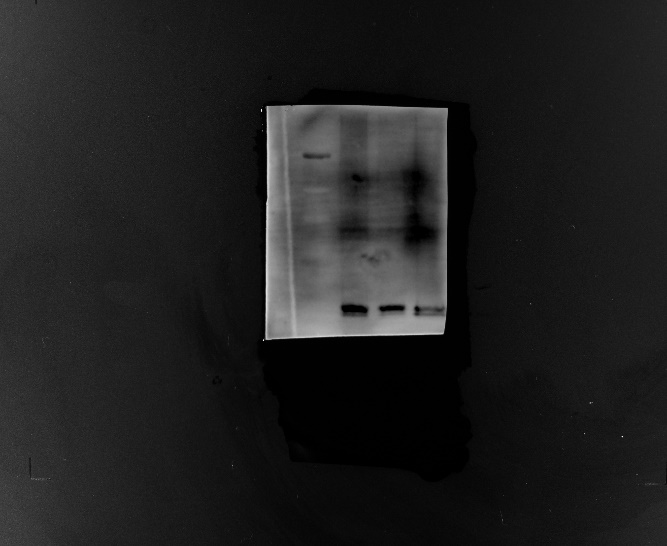 |
| GAPDH: 36kda | 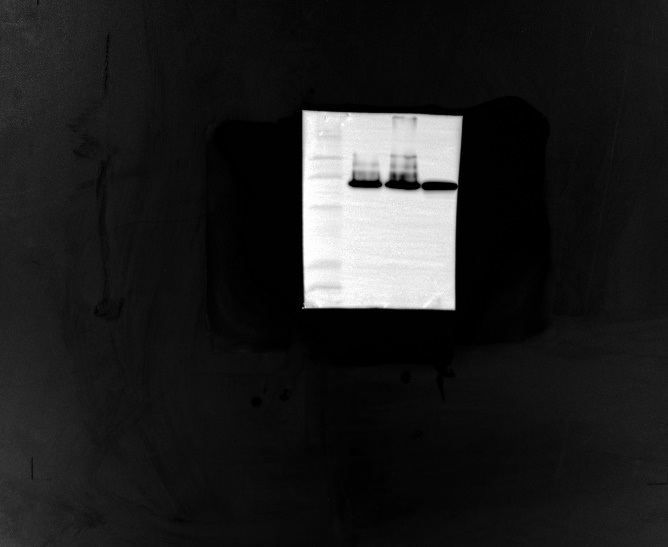 |
