# Supplementary material for: CDC42-mediated Wnt signaling facilitates odontogenic differentiation of DPCs during tooth root elongation
Source: Stem Cell Res Ther. 2023 Sep 19;14:255. doi: 10.1186/s13287-023-03486-2 (PMC10510226; doi:10.1186/s13287-023-03486-2)
Supplement: Supplementary file 4 — Additional file 4: Original blot images of Figure S4C and Figure S4D. [file 13287_2023_3486_MOESM4_ESM.docx]

**Additional file 4.** Original photos of the full bots in Figure4C and Figure4D

| ALP: 70kda | 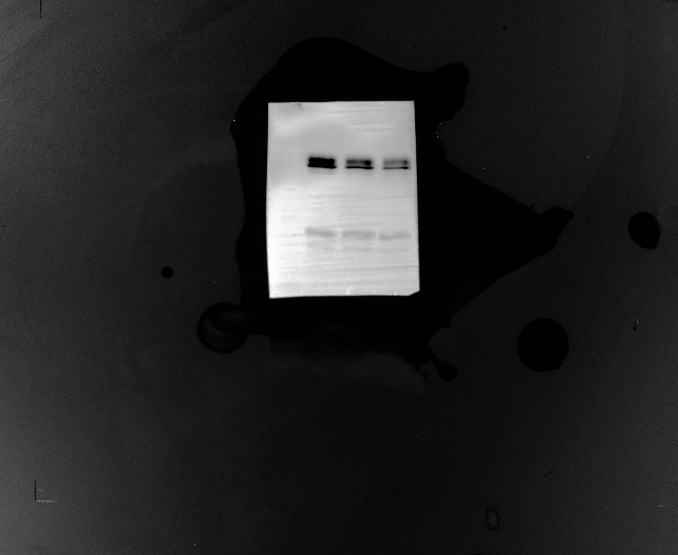 |
| --- | --- |
| COL1: 139kda | 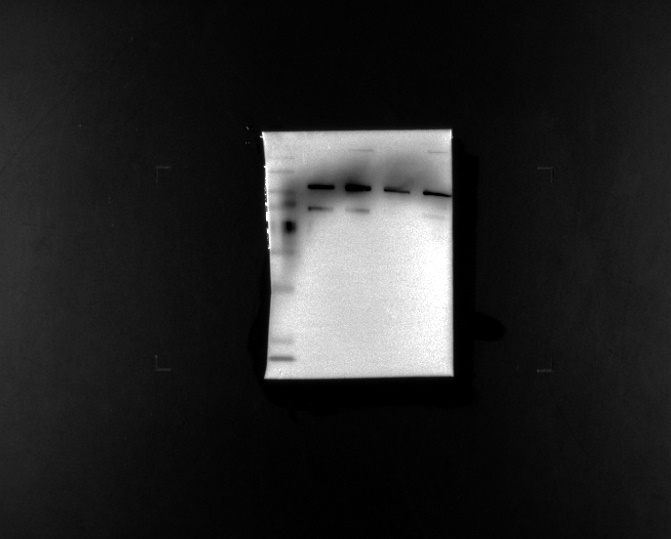 |
| DMP1: 70kda | 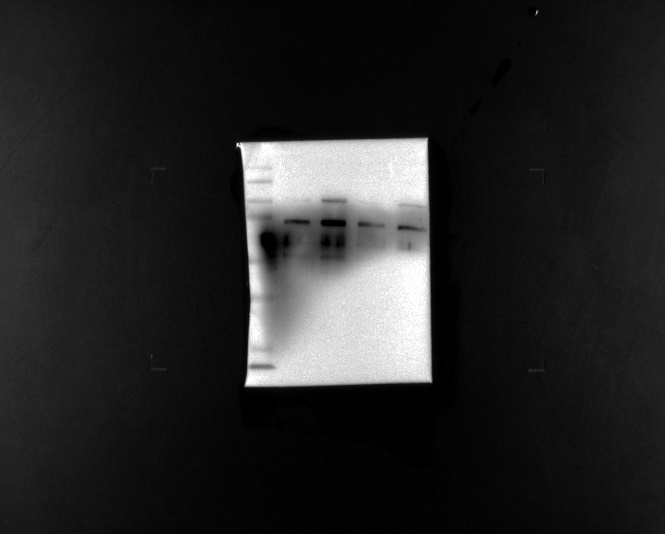 |
| DSPP: 110kda | 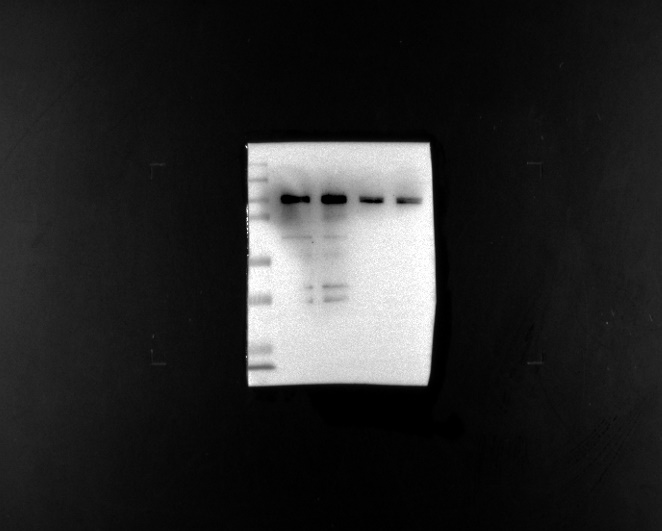 |
| GAPDH: 36kda | 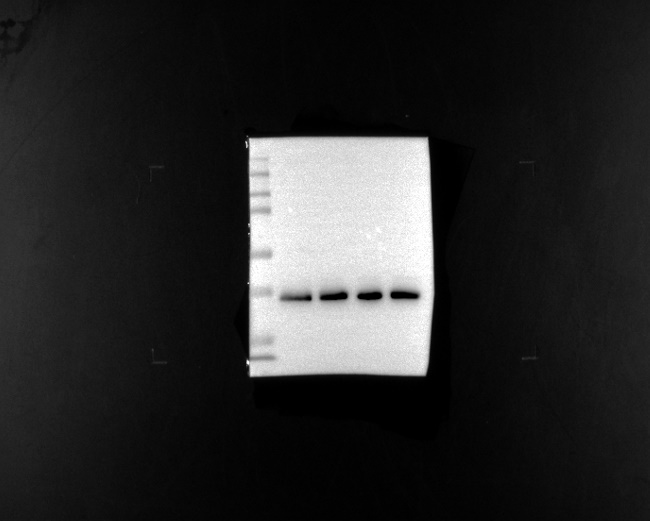 |
| p-GSK3β: 47kda | 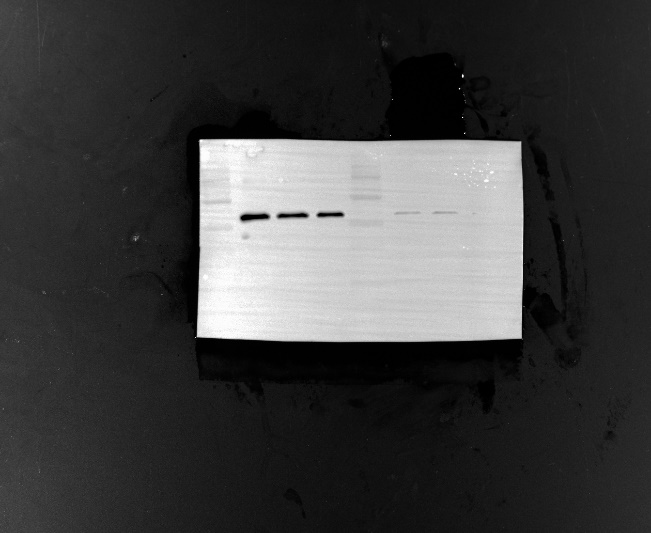 |
| β-catenin: 85kda | 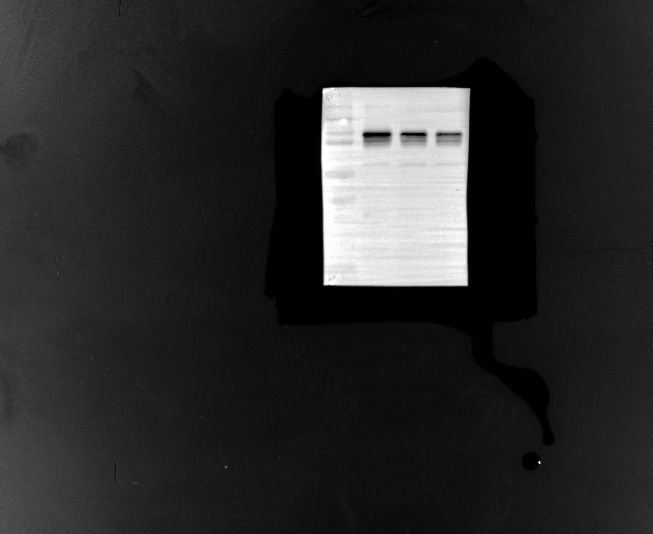 |
| nuclear β-catenin: 85kda | 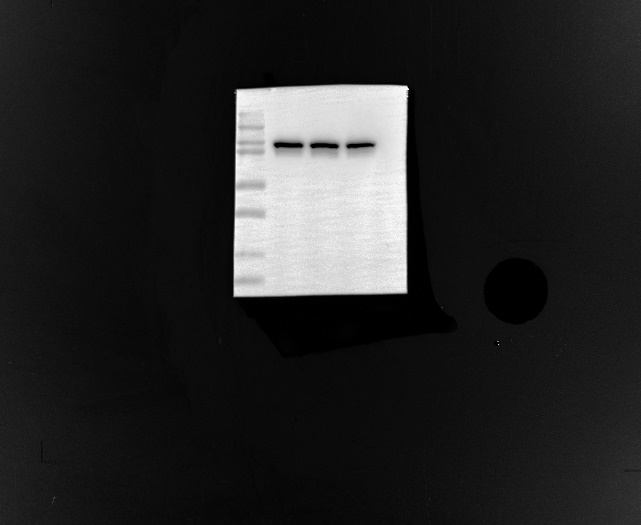 |
| GAPDH: 36da | 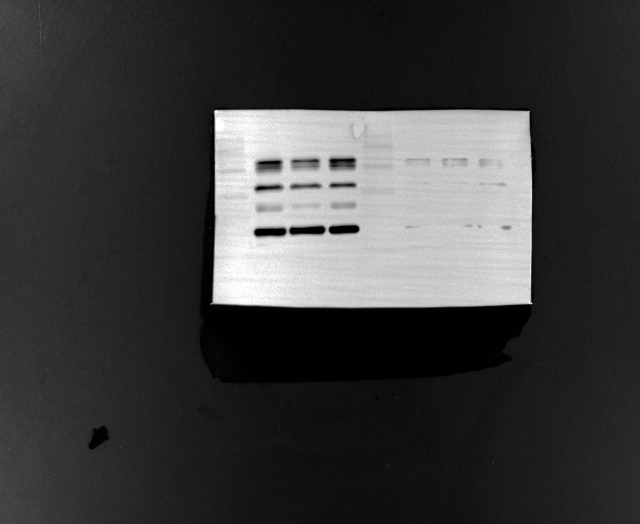 |
